# Smart subsidies for catchment conservation in Malawi

**DOI:** 10.1038/sdata.2018.113

**Published:** 2018-07-03

**Authors:** Andrew Reid Bell, Patrick S. Ward, Lawrence Mapemba, Zephania Nyirenda, Wupe Msukwa, Edwin Kenamu

**Affiliations:** 1 Department of Environmental Studies, New York University, 285 Mercer St., New York, NY, 10003, USA; 2 Environment and Production Technology Division, International Food Policy Research Institute, 1201 Eye St. NW, Washington, DC, 20005, USA; 3 Bunda College, Lilongwe University of Agriculture and Natural Resources, PO Box 219, Lilongwe, Malawi

**Keywords:** Agriculture, Decision making, Developing world

## Abstract

Conservation agriculture (CA) is a management paradigm in which soil is covered outside of cropping seasons, minimally disturbed, and recharged with nitrogen-fixing legumes. Finding effective ways to encourage CA is a centuries-old problem playing out acutely today in Sub-Saharan Africa. To better understand this issue, we have collected data on rural livelihoods and CA adoption during a two-year intervention in southern Malawi. The intervention evaluated rates of CA adoption under two different structures of payment and three levels of monitoring. The dataset includes a baseline and endline survey covering 1,900 households, along with surveys conducted with participants opting into the intervention. Baseline and endline questions included modules on farm-level inputs and production at the plot-crop level; plot characteristics; household composition and assets; savings, loans, and other sources of income; neighborhood characteristics; and perceptions regarding CA. Registration questionnaires in the intervention included detailed assessments of recent production in plots being registered to the intervention, and basic information for all other plots; and basic information on household structure and assets.

## Background and summary

Conservation agriculture (CA) is a suite of land management practices aimed at improving soil structure, with a host of benefits for both the producer and the agricultural landscape. Manifesting itself in slightly different ways across the globe^1^, CA typically includes (i) no or minimal tillage of soils, (ii) retention and mulching of crop residues, and (iii) intercropping or rotation with other crops, usually legumes^2^. Under consistent and sustained adoption of CA practices for a period of several years, soils benefit from increased water retention and improved organic matter, with potential boosts to production for the farmer. At the same time, reduced runoff and soil erosion can provide benefits to water regulation and quality elsewhere in the agricultural landscape^3^.

For Malawi, these cross-scale benefits are particularly important. Malawi’s largely rural economy is heavily invested in subsidized production of monoculture maize^4^ as a means of reducing reliance on food aid or imports^2^, with conventional tillage increasingly resulting in soil erosion with each new season’s rains. In the Shire River Basin, this enhanced sediment loading in riverine systems damages aquatic habitats, threatens farmers’ livelihoods, and dissipates the hydropower potential of dams that provide more than 90 percent of Malawi’s electricity^5^.

CA has been widely promoted throughout Sub-Saharan Africa, but results have been largely disappointing. In particular, adoption rates are frequently as low as only a few percent of total cropped area across the region^1,6^, compared with nearly half of agricultural land under CA in the Americas^1^. Adoption studies have identified many private costs and risks that farmers must bear before benefits of CA might be realized—for example, years of increased weeding, lack of residues for livestock fodder, etc.—as discouraging experimentation, let alone sustained adoption^7,8^. Governmental and nongovernmental agencies interested in promoting CA often resort to providing incentives to farmers to encourage farmers to adopt CA. Yet it is rarely known a priori what magnitude of an incentive would be sufficient to overcome the private costs farmers initially bear, and there is often a great deal of heterogeneity in this regard that may make such approaches highly inefficient. Furthermore, despite years of CA adoption research, there are few universal predictors of CA adoption for Sub-Saharan Africa^9^, highlighting the importance of context and of a deeper look into the decision structure of adoption.

The project from which the current datasets originate involved a two-year evaluation of a particular incentive, with two goals in mind. First, we aimed to undertake the first field evaluation of an “agglomeration payment”^10,11^, an innovative two-part incentive designed to encourage spatial coordination in adoption of conservation practices. It consists of a conventional subsidy (in the form of a voucher that could be used to purchase agricultural inputs through a local input dealer network) in exchange for participation, along with bonus payments for any neighboring farmers who also participate. This creates a range of potential network externalities—heterogeneous pricing as well as the potential for enhanced diffusion, encouragement, and self-policing of compliance—whose impact on CA adoption we wished to evaluate. Second, we wished to use our successive rounds of data collection to examine different aspects of farmer decisionmaking and preferences in order to better elucidate the structure of the adoption decision, both in the presence and absence of specific programs of encouragement, and to better identify contextual factors that encouraged or deterred adoption.

This dataset (Data Citation 1) includes a baseline and endline survey from a cluster-randomized sample of households within villages covering the five uppermost Extension Planning Areas (EPAs) in the Shire River Basin in southern Malawi, spanning the districts of Balaka, Machinga, and Zomba (Table 1). These EPAs were identified by the Malawian Department of Land Resources Conservation (DLRC) as the key riparian areas to the Shire River. Additionally, it includes registration and monitoring data from two years of the experimental intervention, with the intervention consisting of varying incentive structures and visual monitoring of land management practices. Finally, the data include an accounting of what, if anything, recipients purchased with the voucher they received. In sum, these data include detailed assessments of farm and household characteristics; detailed crop-level measurements of inputs and production for all plots (baseline and endline surveys) or the main plot (intervention registrations; summaries at plot-level for all other plots); assessments of access to services, coupons, and markets; an economic experiment designed to assess marginal willingness-to-accept a CA incentive (baseline), and the perceived risks associated with CA (year 2 intervention and endline).

## Methods

### Experimental design

The experimental design used in the impact evaluation consisted of 2×3 (2^1^3^1^) full factorial design with two incentive treatments and three treatments corresponding to the intensity with which village-level program compliance is visually monitored. In particular, treatment villages were randomly assigned to receive either a conventional subsidy (consisting of a base voucher payable per 0.1 acre registered in the program, up to a maximum of 1 acre) or an agglomeration payment (consisting of a base voucher payable per 0.1 acre registered in the program, up to a maximum of 1 acre, plus a bonus payment for each of the registrant’s neighbors who also adopted the set of CA practices, up to total of four neighbors). At the time that villages were randomly allocated to these payment treatment arms, the monetary values of the base voucher and the agglomeration bonuses were not known. As will be discussed in greater detail below, these amounts were determined based on analysis of data from a discrete choice experiment that allowed the research team to ascertain each individual farmer’s willingness-to-accept a subsidy to encourage full compliance with the CA program. Treatment villages were also randomly assigned to receive either full monitoring (in which all registered participants would receive a follow-up visit to their registered plot to verify land management practices), partial monitoring (in which half of registered participants within a village would be randomly selected for a follow-up visit to their registered plot to verify land management practices), or no monitoring (in which no registered participants would be subject to follow-up visits to verify land management practices). Comparison villages were not eligible to receive vouchers as a part of this program, nor were they subject to follow-up visits to verify their land management practices; these villages were visited only as part of the baseline and endline survey efforts. Nevertheless, because of the large number of governmental and non-governmental agencies active in promoting CA in Malawi—including through the use of subsidies or vouchers—we were unable to prevent contamination and ensure strict controlled conditions. To our knowledge, no comparable efforts to encourage CA via incentive occurred in our study area during the study period, with government funding largely focused in the Farm Inputs Subsidy Programme (FISP), which since 2005 has offered coupons for fertilizer and seed to the ‘productive poor’ at a cost of around 10–15% of Malawi’s national budget^12^. Our datasets provide an opportunity ex-post to account for any other incentive programmes present through the baseline and endline modules on coupons received and used; coupon use for fertilizer and seed is similar across control and treatment villages, showing similar benefits from FISP (coupons for maize seed used by 28% of respondents in control and 28% of respondents in treatment villages; for chitowe fertilizer (NPK+S, with 23:21:0+4), use by 40 and 37%, respectively; and for urea fertilizer, use by 41 and 39%, respectively); by contrast, use of flexible vouchers of the kind offered in our intervention was strikingly different, with 11% of respondents in control villages using such coupons with an average value of 550 Malawi Kwacha (~0.75USD) and 30% of respondents in treatment villages using coupons with an average value of 3,100 Malawi Kwacha (~4.5USD).

**Figure 1:**
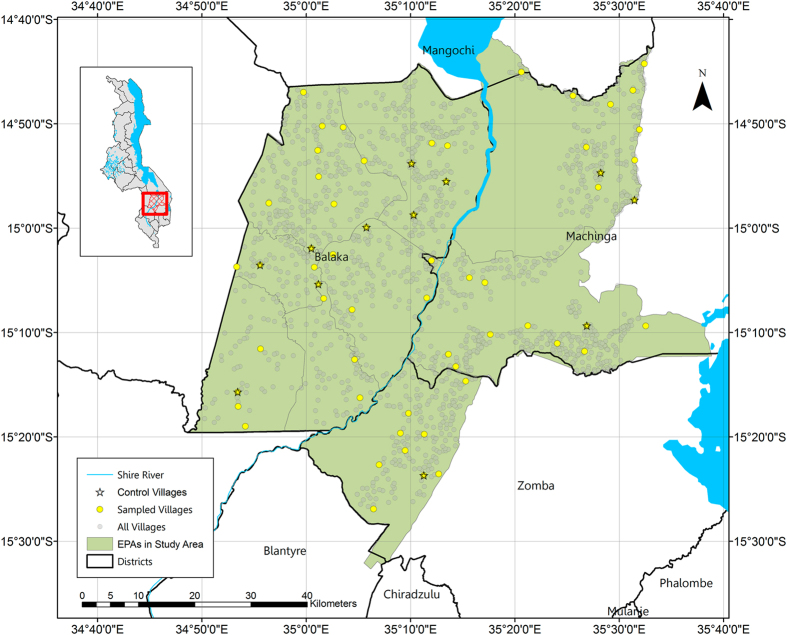
Study area.

To identify our sample villages, we first obtained a list of all villages registered in the pre-selected EPAs from Malawi’s National Statistics Office (NSO). We next wrote an algorithm to generate a large number (1 million) of 60-village simple random samples from this combined list of villages. Our algorithm then selected the sample for which the smallest distance between any two sampled villages was maximized in an attempt to reduce the risk of cross-village contamination. We also used an algorithm to generate a list of the eight nearest villages to each sampled village to serve as alternates. For each village in the sample list, we obtained a list of farming households from the District Agricultural Development Offices (DADO). From this refined list we drew a random sample of 30 households from each village to participate in the study, for a total of 1,800 households (though only 1,791 household records were retained at baseline). In five of the 60 instances, the sampled village name was incorrect—the village had nucleated since the preparation of the NSO list—and we selected the nearest alternate to each village from the list of alternates. Following the selection of these 60 villages, we randomly allocated the villages to one of seven experimental arms: the six treatment arms consisting of different combinations of the two incentive structure factors and the three land management monitoring factors, and the comparison arm (Figure 1).

### Ethical approval

Prior to the initiation of the project—including data collection and implementation of the experimental interventions—the entire research design and protocol were subject to an ethical review conducted by the Institutional Review Board (IRB) at the International Food Policy Research Institute (IFPRI). IFPRI’s IRB is registered with Federalwide Assurance number FWA00005, and is designated IRB number 00007490. Pursuant to the minimum risk review governing the research activities, IFPRI’s IRB ensured that the research team had proper procedures in place to maintain participants’ privacy and the confidentiality of their personal details, as well as ensuring that participants were well-informed of their rights as research subjects prior to consenting to participation.

### Project and survey implementation

**Figure 2:**
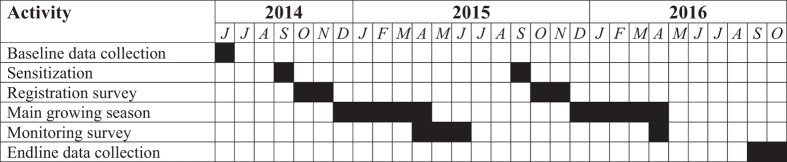
Project Timeline.

The timeline for implementation of project activities is shown in Figure 2. Project activities began in June 2014 with the implementation of the baseline household survey. Among other features, the baseline survey provided us with a broad characterization of households’ familiarity and experience with CA principles—and the three constituent practices—prior to the introduction of the various incentive mechanisms described above. The baseline data were collected by an enumeration team consisting of 20 graduate students from Bunda College of the Lilongwe University of Agriculture and Natural Resources (LUANAR), with surveys conducted via computer-assisted personal interviewing (CAPI) technologies on the CSPro data entry platform.

Importantly, the baseline survey also included a discrete choice experiment that would provide the basis for the base voucher and agglomeration bonus payments that would be administered to treatment households^13^. Discrete choice experiments are a form of stated choice experiment, where preferences are elicited based on responses to hypothetical scenarios rather than observed purchasing decisions. In a discrete choice experiment, individuals are presented a series of choice scenarios in which they must choose between bundles containing different traits (in this case, cultivation and/or land management practices), each taking one of a number of pre-specified levels (such as a binary adoption indicator). Through statistical analysis of participants’ choices given the alternatives available in each choice scenario, the researcher is able to estimate marginal values (in either utility or monetary terms) for the various attributes embodied in the alternatives. The methodology is particularly useful for eliciting valuation of products for which there is not a functioning market in which to observe such revealed preferences, which makes it a particularly useful methodology for analyzing preferences for hypothetical goods and services and for analyzing the welfare effects of multidimensional policy changes.

In our discrete choice experiment, farmers’ were presented with choice scenarios that reflected different agricultural practices required by a given program implementer. Specifically, the attributes included in our choice sets included whether the program required intercropping (yes/no), whether the program required zero tillage (yes/no), the percent of crop residues required to be retained and mulched (as a percentage of total crop residues), who is implementing the program (Total LandCare, TLC; the National Smallholder Farmers’ Association of Malawi, NASFAM; DLRC; or World Vision, WV), and the subsidy amount provided to incentivize the adoption of the package.

**Figure 3:**
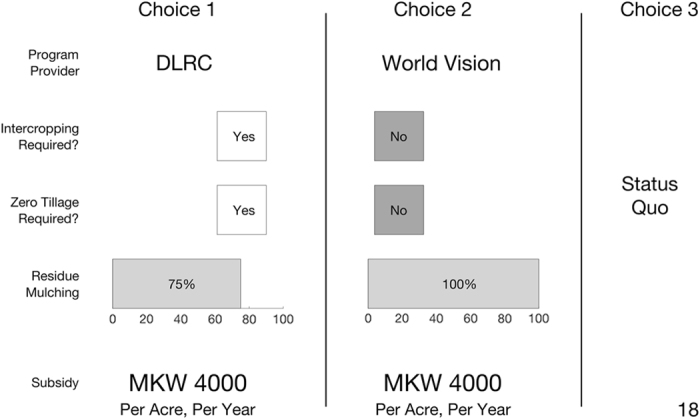
Sample choice set from Baseline Survey DCE.

We constructed a D-Optimal experimental design controlling for all main effects as well as some key two-way interaction effects. D-optimal designs minimize the D-error of the design, computed as the weighted determinant of the variance-covariance matrix of the design, where the weight is an exponential weight equal to the reciprocal of the number of parameters to be estimated. The experimental design was based upon a linear (in the parameters) utility specification with null priors. This design generated 20 unique choice sets that were subsequently randomly assigned to farmers in groups of 10 choice sets each. The random assignment was accomplished by first randomly allocating these 20 unique choice sets into blocks of 10 and then randomizing the order with which each farmer was presented the choice sets in the actual experiment, so as to eliminate any potential order effects. Farmers were randomly allocated to each of these two blocks, with a balanced number of farmers assigned to each of the two blocks. Each choice set contained two alternative hypothetical production practices as well as a status quo (i.e., the production practices used in the most recent agricultural season). An example of a choice card is presented in Figure 3, with attributes of (from top to bottom) provider, requirement for intercropping or rotation, requirement for zero-tillage, percent of crop residues to be mulched on plot, and the value of the subsidy per acre (English version shown; Chichewa translation used in fieldwork).

Analysis of the choice experiment data proceeded immediately following the baseline data collection efforts, as these were to provide the basis for the conventional voucher and agglomeration payment structures. Based on this analysis, it was determined that the conventional voucher in the first year of the program would consist of a payment of MKW 1,200 per 0.1 acre, up to a maximum of 1 acre total, while the agglomeration payment in the first year would consist of a base voucher of MKW 600 per 0.1 acre, up to a maximum of 1 acre total, plus a bonus payment of MKW 200 for each neighbor’s plot undertaking the same set of CA practices as the registrant, up to 4 neighbor’s plots.

Sensitization meetings were organized in each of the treatment villages during September 2014 and 2015. In the sensitization meetings, officers from NASFAM and DLRC explained the program, with a particular emphasis on describing CA and its constituent practices, as well as the short- and long-term benefits of CA for improved soil structure. In treatment villages allocated to receive the conventional voucher, these officers described payment mechanism and how the vouchers could be redeemed for agricultural inputs. Similarly, in treatment villages allocated to receive the agglomeration payment, these officers described how this payment structure would operate, highlighting the additional money that could be earned for farmers who convinced their neighbors to adopt the same sets of CA practices. Villages were also informed of the nature of the program compliance monitoring that registrants in the village would be subject to, though they were not informed that other villages may have different levels of monitoring.

As these sensitization meetings were drawing to a close, a lead farmer from each village would be nominated to maintain a tentative list of villagers wishing to enroll in the program. While these lists were not binding, they served to provide reliable estimates for the number of registrants. These lead farmers were contacted several days prior to registration in order to gauge likely participation, which helped the implementation team in planning for the number of field officers from NASFAM and DLRC to send to each village to assist in the registration activities, which took place in October and November 2014 and 2015. In addition to the field officers from NASFAM and DLRC, the registration teams also included students from the Lilongwe University of Agriculture and Natural Resources (LUANAR). Farmers interested in participating in the program would register specific plots—providing specific data regarding the plot area, altitude, the presence of crop residues, the estimated value of the main crop, soil type, the extent and cause of soil erosion, etc.—while at the same time providing information on basic household details and prior-year land management or conservation practices undertaken. Registration data were recorded manually in logbooks by the field officers from NASFAM and DLRC, while the LUANAR students were responsible for transcribing these data into electronic form using CSPro. Any villager in the treatment villages were eligible to register their plots to participate in project, and there was no requirement that participating in the baseline survey compelled households to subsequently register their plots to practice CA, so there is not a one-to-one correspondence between households participating in the baseline survey and those that registered for participation in the program.

The primary agricultural season in much of Malawi lasts from December until April. This is when Malawi receives the vast majority of its annual rainfall (roughly 95 percent). There were no project activities that occurred during this period, as farmers were typically occupied with agricultural activities.

From April-June 2014 and again in April 2015, following the culmination of this agricultural season, interviewers (20 graduate students from Bunda College) re-visited registrant households to conduct project monitoring. At this time, enumerators also visually verified program compliance (i.e., that farmers followed through with the three CA practices that were required as part of the program) for all registered farmers in the villages randomly assigned to receive full monitoring and for a random sample of half of registered farmers in the villages randomly assigned to receive partial monitoring. For these farmers, payments (whether the conventional voucher or the agglomeration payment) were based on these visual assessments. For the other half of registered farmers in the partial monitoring villages and for the farmers in the no-monitoring village, documented compliance with the program requirements and subsequent voucher payments were based on self-reported land management practices. Both the 2014 and 2015 monitoring surveys were implemented using CSPro. Vouchers were redeemable as cash toward inputs purchases at our private-sector partner Agora agro-dealers, who collected logs of redeemed vouchers and the purchases they were applied to when they were redeemed.

The endline household survey was conducted during September and October 2016. It was initially intended that all households initially visited as part of the baseline survey would be re-visited during the endline interviews, though because of various sources of respondent attrition, we were only able to located 1,565 of the original 1,791 households that were interviewed at baseline, representing a 12.6 percent attrition rate. The enumeration team at endline consisted of 20 graduate students from Bunda College (LUANAR). Unlike previous surveys, which had utilized CSPro for data entry, the endline survey relied on the SurveyCTO CAPI architecture, which is based on the open-source CAPI tool Open Data Kit (ODK). Virtually all of the questions that were asked during the baseline survey were replicated during the endline survey, providing the opportunity for intertemporal comparisons and fairly clean identification of the village-wide effects of the experimental treatments (i.e., intention-to-treat effects).

## Data Records

The data described here are stored in the data repository (Data Citation 1) as standalone databases, though there are opportunities for merging and/or appending data to facilitate richer analyses. In particular, the baseline and endline can be appended to form a two-period panel. The Year 1 Registration and Monitoring data are published as merged, as are the Year 2 Registration and Monitoring data. These Year 1 and Year 2 data can be appended, and in any cases where households registered in both Year 1 and Year 2, they share the same identifier across both years. Ultimately, the voucher utilization data can also be appended to the Year 1 and Year 2 registration and monitoring data. While it is likely as well that some individuals randomly selected for the baseline and endline surveys also opted to participate in the intervention, this is not tracked directly via an identifier. However, participation is indicated by households responding that they had been offered incentives to adopt CA (variable pc51 in Endline) and had received flexible use vouchers (variable cu02_name in Endline).

## Technical Validation

Our sampling design (random sampling of pooled list of villages across study area, and random sampling of households within villages for baseline and endline) coupled with our village sampling method (maximizing the minimum separation of sampled villages across a large number of potential samples), was our best effort at maximizing explanatory power for the region while minimizing risks from John Henry effects (awareness among participants in different treatments or control that other participants’ benefits are different, thus shifting behavior). Additionally, we took great care in structuring outcomes of interest in our baseline and endline surveys (such as land management practices), to decouple them from our intervention and minimize Hawthorne effects (shifts in behavior among participants due only to perception of being observed). Our sampling method also minimizes the risk of between-sampled village spillover effects, while within-sampled village spillover effects (on adoption of conservation agriculture) are in fact encouraged by the design of our incentives.

Errors in data collection were minimized by retaining the same field management staff throughout the duration of the project and re-engaging the same set of graduate students to the extent possible along different periods of data collection, with one-week training and pre-test sessions completed for each round. Further, the use of CAPI software such as CSPro and ODK minimizes risks of improper data capture through the use of logical checks on data entry at the time survey items are asked, and eliminates the process (and associated errors) from data transcription and entry from paper surveys.

## Usage Notes

We describe here notes for understanding final record counts and connections across the datasets.

Firstly, the final count of villages in the intervention and endline datasets is 63, rather than the design count of 60. Malawian villages change and move regularly, and in several instances during our study, a team went to offer the incentive in a village close by and with a similar name to our sampled village. Our protocol was to retain the new villages in the intervention and endline, while returning as well to the originally sampled village. These new villages have no baseline data. Secondly, where households from the baseline study could not be contacted, additional households were added to the endline to maintain at least 30 households per village.

Lastly, as our intervention included treatments in which not all (or no) respondents received a follow-up field visit to verify their compliance. Compliance is being verified by remotely sensed images along with plot boundary data collected at the time of registration. These data are not included as part of the datasets above, but may be obtainable from the study authors with appropriate IRB protocols submitted at the requestor’s home institution; please contact the authors directly to discuss.

## Additional information

**How to cite this article**: Bell, A. R. *et al*. Smart subsidies for catchment conservation in Malawi. *Sci. Data* 5:180113 doi: 10.1038/sdata.2018.113 (2018).

**Publisher’s note**: Springer Nature remains neutral with regard to jurisdictional claims in published maps and institutional affiliations.

## Supplementary Material



## Figures and Tables

**Table 1:** Dataset summary.

Survey round	Selection criteria and size
Baseline Survey, including embedded Discrete Choice Experiment	Cluster-random sample of households (~30) within villages (60), for a total of ~1,800 households
Year 1 Intervention Participant Registration and Monitoring Survey, and Voucher Use Log	Opt-in participation by households in randomly selected villages (48), totaling ~1,450 households
Year 2 Intervention Participant Registration and Monitoring Survey, and Voucher Use Log	Opt-in participation by households in randomly selected villages (48), totaling ~2,800 households
Endline Survey	Cluster-random sample of households (~30) within villages (63 – See usage notes) for a total of ~1,900 households
